# A comparative analysis of the outcome of unilateral laminotomy and conventional laminectomy in patients with single-level degenerative lumbar spondylolisthesis: a single-center retrospective study 104 patients on postoperative pain and functional disability

**DOI:** 10.3389/fsurg.2025.1661398

**Published:** 2025-10-23

**Authors:** Yan Li, Congcong Zhu

**Affiliations:** 1 Department of Orthopedics, Sixth People’s Hospital, Chengdu, Sichuan, China; 2 Medical Department, Sixth People’s Hospital, Chengdu, Sichuan, China

**Keywords:** unilateral laminotomy, conventional laminectomy, single-level degenerative lumbar spondylolisthesis, bilateral decompression, clinical outcomes, radiologic outcomes

## Abstract

**Objective:**

To compare the clinical and radiologic outcomes of unilateral laminotomy vs. conventional laminectomy for decompression in patients with single-level degenerative lumbar spondylolisthesis.

**Methods:**

This retrospective study included 104 patients who underwent decompressive surgery for single-level degenerative lumbar spondylolisthesis at a single institution. Clinical outcomes were assessed using the Oswestry disability index (ODI) and the visual analog scale (VAS) for back and leg pain. Radiologic outcomes were evaluated by measuring translational motion, disc height, and epidural cross-sectional area on imaging.

**Results:**

The average age of patients was 58.95 years (range: 40–79). Both groups showed comparable VAS scores for lamb pain and complication rates. However, the unilateral laminotomy group had significantly lower VAS scores for back pain and ODI scores within the group. Improvements in these scores were more pronounced in the unilateral laminotomy group compared to the conventional laminectomy group, reflecting within-group comparisons. The unilateral laminotomy group also experienced less intraoperative blood loss and shorter surgical time. Radiographically, there were no significant differences (*P* > 0.05) in translational motion or disc height between the two groups. However, the traditional laminectomy group showed a larger epidural cross-sectional area. Postoperative complications were rare.

**Conclusions:**

Both unilateral laminotomy and conventional laminectomy provide effective pain relief and adequate decompression for spinal stenosis. Unilateral laminotomy offers the advantage of reduce blood loss, shorter operative time, and lower VAS scores for back pain. Radiographically, the two procedures produce similar outcomes in terms of translational motion and disc height, although the traditional laminectomy group exhibited a larger epidural cross-sectional area. Despite this, VAS scores for leg pain were comparable between the two groups. Further studies are needed to compare the effects of surgical techniques at different lumbar levels.

## 1 Introduction

Degenerative lumbar spondylolisthesis frequently coexists with adjacent segments lumbar multilevel stenosis (MSS) (1). Surgical treatment for degenerative lumbar spinal stenosis typically involves decompression of the neural structures, either with or without spinal fusion. A conventional laminectomy which removes the posterior bony and ligamentous components, provides adequate decompression while preserving the facet joints and other stabilizing structures of the spinal segment. However, iatrogenic instability may arise from compromised mechanical integrity during extended decompression procedures, potentially leading to significant symptoms. Additionally, decompression can exacerbate pre-existing instability in certain patients, particularly those with degenerative spondylolisthesis. In such cases, spinal is often performed to eliminate the risk of further instability and to prevent disease progression (2).

The evolution of minimally invasive spine surgery has led to smaller incisions, aiming to minimize iatrogenic instability. Microscopic laminotomy, a minimally invasive technique, has demonstrated success in performing bilateral decompressions of spinal stenosis using both unilateral laminotomy and conventional laminectomy. This suggests that the traditional laminectomy approach may eventually be replaced by more minimally invasive techniques (3). Laminotomy, as a less invasive procedure, has the advantage of preserving more of the spinal structures, including the facet joints and the ligamentous complex, which are important for spinal stability. In contrast, conventional laminectomy involves the removal of more bony and ligamentous structures, which may increase the risk of postoperative instability and complications, such as muscle atrophy and loss of spinal stability. However, laminectomy provides a more thorough decompression, especially in cases with severe stenosis or multilevel disease, making it a preferred option in such scenarios. The choice between laminotomy and laminectomy often depends on the severity of stenosis, the degree of spinal instability, and the surgeon's preference.

At the index surgery level, a unilateral laminotomy preserves contralateral tissues, including the lamina and facet joint, theoretically maintaining more spinal stability compared to conventional laminotomy. Consequently, unilateral laminotomy offers a potential structural advantage that may help to sustain spinal stability (4–9). However, no studies to date have conclusively demonstrated this advantage when compared to bilateral laminotomy, particularly in patients with lumbar spondylolisthesis (10–13). Two comparative studies focused on multilevel procedures and short-term follow-up did not address postoperative stability but reported superior clinical outcomes and fewer complications for bilateral laminotomies.

Given these findings, the aim of this study is to compare the clinical and radiological outcomes following unilateral and bilateral laminotomies for the treatment of single-level lumbar spondylolisthesis (14).

## 2 Materials and methods

### 2.1 Patients

A retrospective analysis was conducted on 110 patients who underwent bilateral decompression for lumbar spondylolisthesis via unilateral laminotomy or conventional laminectomy at The Sixth People's Hospital of Chengdu between 2017 and 2022. The inclusion criteria were as follows: patients with single-level degenerative lumbar spondylolisthesis, low back and leg pain unresponsive to conservative treatment for at least three months, presence of central canal stenosis, and dynamic radiographs showing instability and grade one degenerative spondylolisthesis. After applying these inclusion criteria, a total of 110 patients were identified.

Exclusion criteria included: a history of prior spine surgery, spinal infections, spinal instability not meeting the inclusion criteria (e.g., severe instability, as defined by unacceptable sagittal or coronal malalignment), and spinal fractures. Six patients were excluded from the study for the following reasons:

1. Insufficient radiological data (3 patients),
2. Inadequate follow-up (3 patients).

This left 104 patients who met all inclusion criteria and provided complete data for the study.

The surgery was performed by two experienced surgeons, and the patients were divided into unilateral laminectomy group and traditional laminectomy group according to the surgical approach. All patients had single-level degenerative lumbar spondylolisthesis, with low back pain and leg pain persisting for at least three months, unrelieved by conservative measures. Central canal stenosis was present in all patient. Both the unilateral laminotomy and conventional laminectomy group exhibited comparable clinical symptoms and radiographic stenosis severity at the time of surgery.

The inclusion and exclusion criteria were as follows:

Inclusion Criteria:

1. Patients with single-level degenerative lumbar spondylolisthesis.
2. Low back and leg pain unresponsive to conservative treatment for at least three months.
3. Presence of central canal stenosis.
4. Dynamic radiographs showing grade one degenerative spondylolisthesis and assessing spinal instability. Flexion-extension lateral radiographs were used to measure sagittal instability in the spine.

Exclusion Criteria:

1. History of prior spine surgery, including spinal fusion or revision surgery. Patients with significant comorbidities, such as diabetes, hypertension, or other conditions that could significantly affect postoperative outcomes.
2. Spinal infection.
3. Spinal instability not meeting the inclusion criteria (e.g., severe instability, as defined by unacceptable sagittal or coronal malalignment). For clarity, spinal instability was defined as excessive translation or angular changes in vertebral alignment, exceeding the threshold of normal movement as outlined in previous studies.
4. Spinal fractures.

In addition to sagittal instability assessed by dynamic radiographs, whole-spine radiographs (standing) were considered for a complete assessment of both sagittal and coronal alignment. Measurements of sacral slope and plumb line were used as part of the comprehensive evaluation of spinal stability, recognizing that degenerative changes in the lumbar spine do not occur in isolation and must be considered in the context of the entire spine.

This study was reviewed and approved by the hospital ethics committee (Approval number: ZCKT-2020-0002).

### 2.2 Surgical method

Both surgical techniques were performed using a standard midline approach under general anesthesia. In the unilateral laminotomy group, the supraspinous and interspinous ligaments were preserved throughout the procedure. For the conventional laminectomy group, the interlaminar space was exposed following the dissection of the paraspinal muscles from the midline after making skin and fascial incisions. In the unilateral laminotomy procedure, a laminotomy was performed under microscopic visualization. This involved the removal of a portion of the medial facet and a small section of the superior and inferior laminae at the target segment. The supraspinous and interspinous ligaments were preserved. AKerrison punch was used to excise the ligamentum flavum and its bony attachments, exposing the dural sac. After the initial laminotomy on one side, the operating table was tilted contralaterally and the microscope was angled toward the medial side to facilitate further decompression on the contralateral side. The deep cortical surface of the contralateral lamina was removed using a burr, and decompression of the contralateral lateral recess and foramen was performed. The ligamentum flavum and its bony attachments on the contralateral side were also removed. Adequate decompression of the nerve roots on both sides was confirmed before closure. In the conventional laminectomy procedure, the interlaminar space was similarly exposed, and a complete laminectomy was performed by removing the lamina, facet joints, and ligamentum flavum. This approach aimed to achieve optimal decompression of the neural elements. Following decompression, an interbody fusion device was inserted, and bilateral screws were placed to stabilize the intervertebral space. Fusion success was defined as the presence of solid bony fusion on postoperative radiographs and CT imaging, with no motion at the surgical site. Pseudarthrosis was diagnosed if there was evidence of non-union on imaging despite the clinical absence of fusion. Uncertain fusion was defined as incomplete or questionable fusion based on imaging findings (x-ray and CT). A final check for adequate decompression and stability was performed, ensuring no further compression of the neural structures. Pressure was applied to close the wound securely. Postoperatively, all patients were allowed to walk twelve hours after surgery, and typically, they were discharged on the third postoperative day. A six-week immobilization period using a soft corset was followed by a structured rehabilitation program that included back strengthening exercises.

### 2.3 Observation indicators

Clinical and radiologic follow-ups were conducted at three months post surgery. Clinical outcomes were assessed using the Oswestry disability index (ODI) (15) and the Visual Analog Scale (VAS) (16) for back and leg pain. These measurements were recorded at each follow-up visit by an impartial observer who was blinded to the group allocation of the patients. The mean improvement scores were calculated by subtracting the preoperative values from the postoperative scores at each time point.

Radiologic measures were evaluated by two independent physicians who were blinded to the clinical information of the patients, ensuring unbiased assessment. Digital tools in the Picture Archiving and Communication System (PACS), PiView 1.0 (Infinitt Co. Ltd., Seoul, Korea), were used for the analysis. All patients underwent preoperative imaging, including plain radiographs (with dynamic views), computed tomography (CT), and magnetic resonance imaging (MRI). Postoperatively, CT scans were performed at the last follow-up visit, while routine plain radiographs were obtained at each follow-up interval.

### 2.4 Follow-up appointment

**Figure 1:**
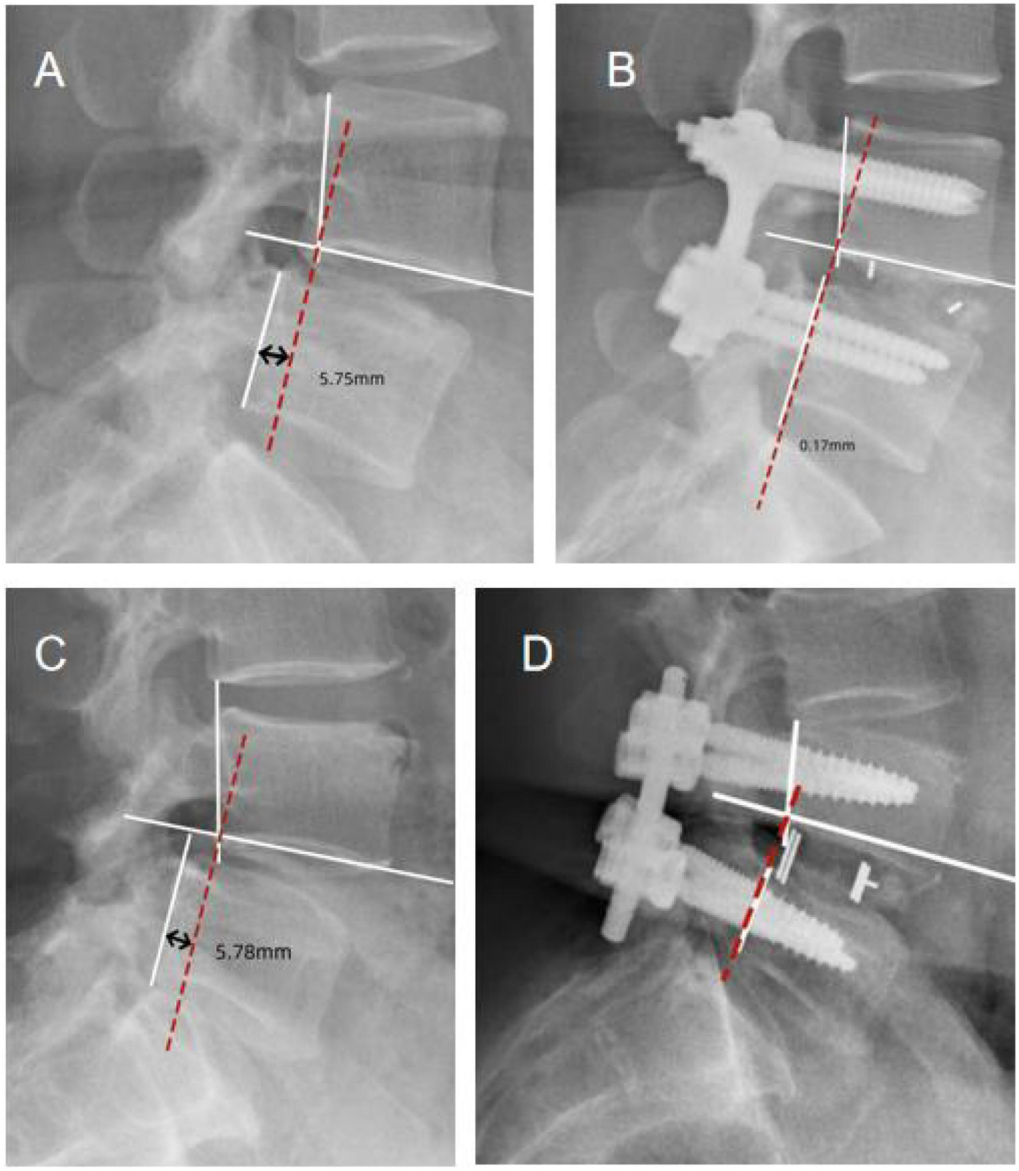
Translational motion measurement. The distance from the posterior vertebral line of L4 to the posterior edge of L5 on the upper endplate of L5 was measured in flexion and extension radiographs. [**(A)** Unilateral laminotomy, Preoperative; **(B)** unilateral laminotomy, Postoperative; **(C)** conventional laminectomy, Preoperative; **(D)** conventional laminectomy, Postoperative].

Radiological outcomes were assessed using digital tools to assess the cross-sectional area of the dural sac at the disc level in preoperative CT scans and in postoperative CT scans obtained at 3months, to evaluate the degree of decompression. Preoperative and postoperative dynamic radiographs were used to analyze translational motion and disc height. Disc height was measured using lateral radiographs, with the height determined by the distance between parallel lines to the superior and inferior vertebral endplates. Translational motion was measured using the technique described by Dupuis et al., measuring the distance between the posterior vertebral line of the superior vertebra and the rear edge of the inferior vertebra, specifically on the upper endplate of the inferior vertebra. To correct for potential radiograph magnification errors, the observed translational distance was normalized to as a percentage of the upper vertebral body width. The translation movement was used as an indicator to assess spinal instability (Figure 1) (17).

### 2.5 Statistical analysis

The variability of numerical data was expressed as the mean ± standard deviations (SD). Statistical analyses were performed using SPSS version 26.0 software. To assess patient demographic characteristics and baseline data, Fisher's exact test and the Chi-square test were applied for categorical variables. For continuous variables, normality testing was performed using the Shapiro–Wilk test before applying *t*-tests, ensuring that the assumption of normal distribution was met for all data analyzed using *t*-tests. In cases where data were not normally distributed, Mann–Whitney *U* test was employed.

Clinical outcomes between the two study groups were compared using the Mann–Whitney *U* test for non-normally distributed data. The Wilcoxon signed-rank test was used to compare preoperative and postoperative clinical outcome scores within each group. Radiological measurements were analyzed using the independent *t*-test for normally distributed data and the Mann–Whitney *U* test for non-normally distributed data. A *P*-value of less than 0.05 was considered statistical significance.

## 3 Results

### 3.1 Demographic and perioperative comparisons

There was no significant difference in patient demographics between the two treatment groups (Table 1). The average age of patients ranged from 40 to 79 years, with a mean age of 58.95 years. The mean follow-up duration was 36.01 weeks (range: 34–38 weeks). Regarding perioperative outcomes, the unilateral laminotomy group had a significantly shorter operation time (55.29 ± 3.65 min vs.89.61 ± 4.68 min, *P* = 0.000) and lower estimated blood loss (150.58 ± 5.12 ml vs.404.62 ± 34.54 ml, *P* = 0.0056) compared to the conventional laminectomy group. There were two perioperative complications in each group. In the unilateral laminotomy group, one patient experienced significant motor weakness in ankle dorsiflexion on the contralateral side immediately after surgery. This weakness gradually improved over several months, with full recovery. In the conventional laminectomy group, one patient development a hematoma eight hours after surgery, which required reoperation due to severe back pain and neurologic deterioration. Two patients in the conventional laminectomy group experienced dural tears, one of which required reoperation due to severe back pain and neurologic deterioration. The neurologic symptoms resolved following the second surgery. Additionally, four patients in the conventional laminectomy group developed postoperative spondylolisthesis, but this was not statistically significant (*P* > 0.05).

**Table 1:** Patient basic information.

Variable	Unilateral laminotomy	Conventional laminectomy	*t/χ*²	*P*
Cases	52	52		
Mean age (years)	59.19 ± 7.74	58.71 ± 8.46	0.292	0.763
Sex			0.559	0.453
Male	20	24		
Female	32	28		
Follow-up period (months)	36.00 ± 1.01	36.01 ± 1.00	−0.092	0.922
Operation time (min)	55.29 ± 3.65	89.61 ± 4.68	−48.948	*P* < 0.001
EBL (ml)	150.58 ± 5.12	404.62 ± 34.54	−48.818	*P* < 0.001
Perioperative complication				
Dural tear	2	1		
Hematoma requiring reoperation	1	0		
Neurological	2	2		
Postoperative spondylolisthesis	0	1		

### 3.2 Clinical outcomes and pain reduction

Postoperative clinical outcomes showed significant improvements in both groups' regarding pain level and disability (all, *P* < 0.05) (Table 2). Preoperative VAS back pain scores were similar for both groups (7.23 ± 0.67 vs.7.23 ± 0.70), but there was a more substantial improvement in the postoperative VAS back pain score in the conventional laminectomy group (4.15 ± 0.75) compared to the unilateral laminotomy group (1.75 ± 0.62). This difference was statistically significant (*P* < 0.001).

**Table 2:** Clinical outcomes.

Outcome	Unilateral laminotomy	Conventional laminectomy	*t*	*P*
Preoperative VAS back	7.23 ± 0.67	7.23 ± 0.70	0.003	1.000
Postoperative VAS back	1.75 ± 0.62	4.15 ± 0.75	−19.324	*P* < 0.001
*P*	0.000	0.000		
Preoperative VAS leg	7.23 ± 0.58	7.25 ± 0.62	−0.163	0.871
Postoperative VAS leg	1.85 ± 0.75	1.79 ± 0.72	0.404	0.691
*P*	0.000	0.000		
Preoperative ODI	33.92 ± 0.74	34.02 ± 0.70	−0.752	0.497
Postoperative ODI	17.13 ± 1.72	23.27 ± 0.82	−22.847	*P* < 0.001

The postoperative VAS leg pain score were similar between the groups, with the unilateral laminotomy group reporting a score of 1.85 ± 0.75 and the conventional laminectomy group scoring 1.79 ± 0.72. However, the difference between two groups was not statistically significant (*P* > 0.05). Regarding the Oswestry Disability Index (ODI), the unilateral score (3.46 ± 0.06) compared to the conventional laminectomy group (5.32 ± 0.11). Although the difference in ODI scores was notable, it was not statistically significant (*P* < 0.001).

### 3.3 Radiographic assessment and decompression effectiveness

**Figure 2:**
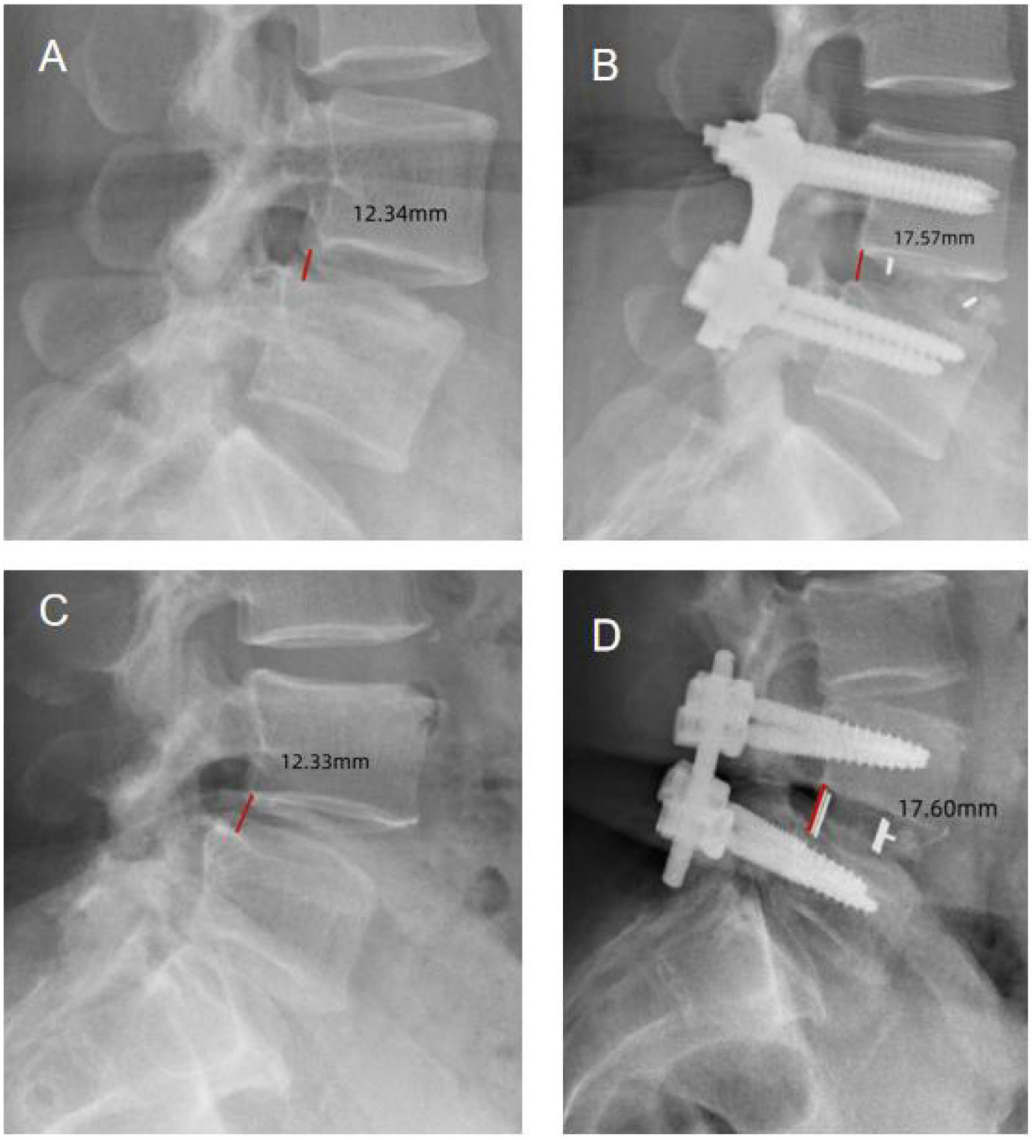
Disk height measurement: the height was measured between the posterior inferior endplate of the upper vertebra and the posterior superior endplate of the lower vertebra. [**(A)** Unilateral laminotomy, Preoperative; **(B)** unilateral laminotomy, Postoperative; **(C)** conventional laminectomy, Preoperative; **(D)** conventional laminectomy, Postoperative].

**Figure 3:**
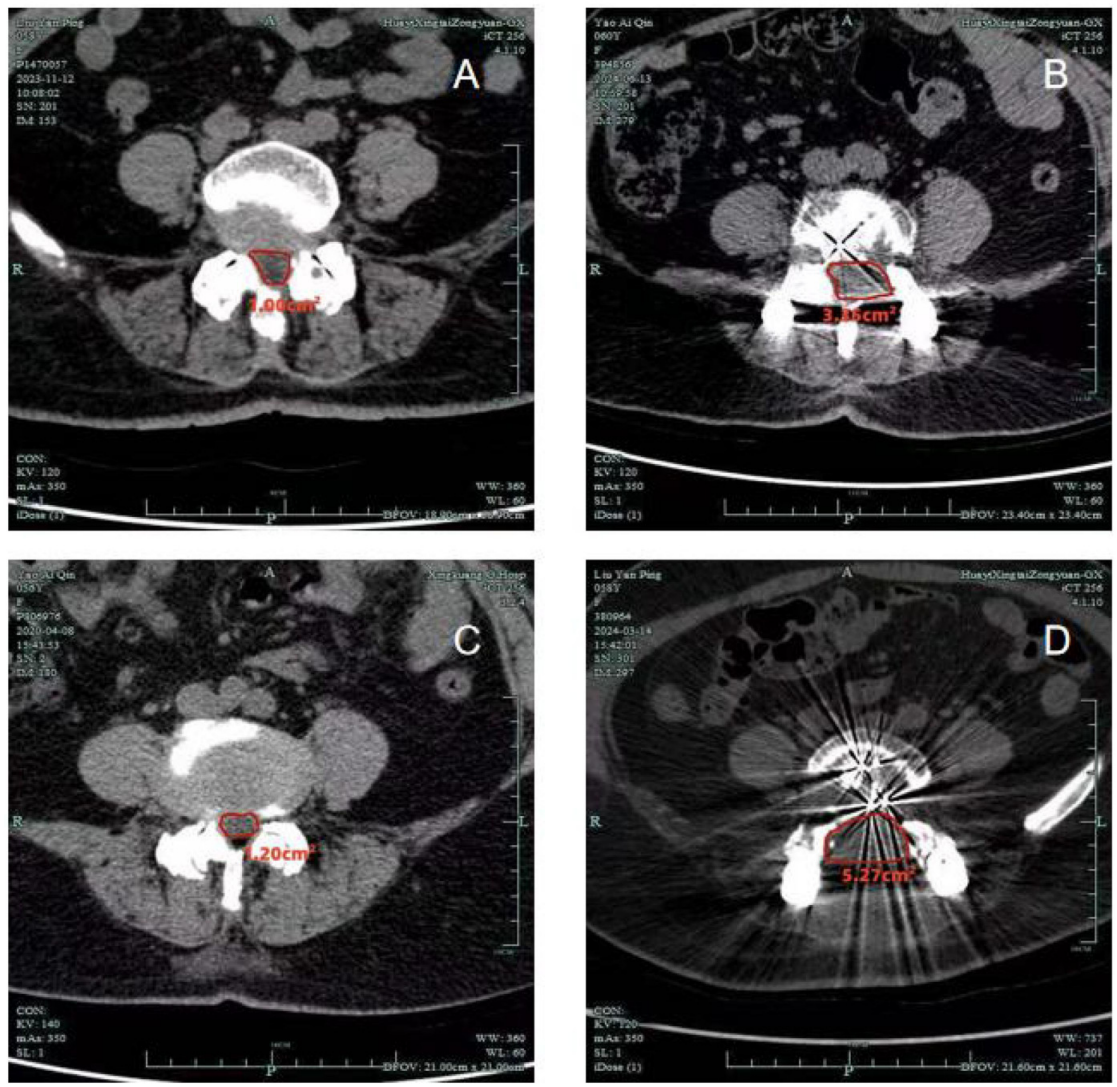
Computed tomographic scans showing the dural sac cross-sectional area, measured using an automated digital tool in the PACS system. [(A) Unilateral laminotomy, Preoperative; **(B)** unilateral laminotomy, Postoperative; **(C)** conventional laminectomy, Preoperative; **(D)** conventional laminectomy, Postoperative].

Radiographic data from both groups are summarized in Table 3. Postoperative CT scans demonstrated that both groups achieved adequate decompression, with no recurrence or residual stenosis observed (Figures 1–3). However, the conventional laminectomy group showed a significantly greater increase in the dural sac cross-sectional area after surgery compared to the unilateral laminotomy group (5.32 ± 0.11 mm^2^ vs. 3.46 ± 0.06 mm^2^, *P* < 0.001). Although the conventional laminectomy group exhibited a significantly larger epidural cross-sectional area, this may not necessarily translate into better clinical outcomes. A larger dural sac area could potentially provide more room for neural structures, which may contribute to better decompression. However, the clinical implications of this finding remain unclear, and further studies are required to evaluate the long-term effects of this difference on spinal stability and postoperative outcomes.

**Table 3:** Radiographic measurements.

Measurement	Unilateral laminotomy	Conventional laminectomy	*t*	*P*
Dural sac cross-sectional area				
Preoperative	1.14 ± 0.11	1.15 ± 0.11	−0.488	0.861
Postoperative	3.46 ± 0.06	5.32 ± 0.11	−62.789	*P* < 0.001
*P*	0.000	0.000		
Translational motion				
Preoperative	5.78 ± 0.28	5.80 ± 0.25	−0.394	0.769
Postoperative	0.16 ± 0.05	0.16 ± 0.05	0.002	0.567
*P*	0.000	0.000		
Disk height				
Preoperative	12.33 ± 0.32	12.32 ± 0.30	0.168	0.827
Postoperative	17.59 ± 0.33	17.61 ± 0.31	−0.349	0.760
*P*	0.000	0.000		

There was no significant difference between the two groups in terms of translational motion or disk height increase (*P* > 0.05). Radiographic instability, characterized by anterior translation, was observed in one patient in the unilateral laminotomy group and four patients in the conventional laminectomy group. Despite this, none of the patients required reconstructive surgery.

## 4 Discussion

The surgical treatment of adjacent-level spinal stenosis in conjunction with mono-segmental instability remains a topic of ongoing debate. While minimally invasive spine surgery (MISS) has gained popularity over the past two decades, many surgeons still advocate for total laminectomy and fusion in these cases. Iatrogenic instability can result from extensive laminectomies performed during traditional procedures (18, 19). The interspinous ligament complex, which plays a crucial role in limiting flexion and axial rotation and providing additional stability, is often comprised during conventional laminectomy. This disruption may increase the likelihood of postoperative instability (20). Resection of the ligamentous complex attached to the spinous processes could accelerate segmental instability.

Some studies have shown that minimally invasive decompression (MIS) does not exacerbate instability to the extent that secondary fusion becomes necessary, even in patients with moderate preoperative spondylolisthesis and stenosis (21). Additionally, segments adjacent to a spinal fusion are more prone to instability following decompression, as they are subject to increased biomechanical load (22–24).

To minimize postoperative instability, less intrusive decompression procedures have been explored. Unilateral laminotomy has been demonstrated to produce effective bilateral decompression outcomes compared to traditional decompression techniques (25–27). However, most of the focus in the existing literature has been on spinal stenosis rather than lumbar spondylolisthesis (14). In this study, we compared unilateral laminotomy and conventional laminectomy for the treatment of single-level lumber spondylolisthesis. We found that while unilateral laminotomy preserves the contralateral articular eminence and the spinous ligament complex, it offers greater stability compares to the bilateral approach.

Several studies have suggested that unilateral laminotomies can produce results as good as, or even better than those of a whole laminectomy (28, 29). During conventional laminectomy, the spinous process, interspinous, and supraspinous ligaments are removed. These ligaments have been shown to withstand up to 19% of flexion pressures, so their removal may compromise flexion stability (30). Unilateral laminotomy, by sparing the middle ligament complex, has been associated with superior clinical outcomes, especially in patients with multilayer stenosis (31). Contrary to the belief that severe central spinal stenosis necessitates a full laminotomy, unilateral laminotomy can often provide adequate decompression (32).

Conventional laminectomy is a straightforward procedure that can be performed with or without microscopic visualization. However, it requires paraspinal muscle retraction, partial removal of the facet joints, and excision of the midline ligament complex. Unilateral laminotomy, by contrast, may cause less damage to the spine's supporting tissues. It is well recognized that postoperative muscle retraction during surgery can lead to electromyographic anomalies and postoperative muscle atrophy (33, 34). One major factor contributing to suboptimal outcomes following spinal stenosis decompression is denervation of the back muscles and paraspinal muscular atrophy (35). By preserving the contralateral facet joint, unilateral laminotomy may avoid some of these complications. Several studies have similarly reported that unilateral laminotomy is associated with shorter operative times compared to conventional laminectomy. For instance, a study by Ko et al. (36) found that unilateral laminotomy reduced surgical time by approximately 30%, while also resulting in lower blood loss and fewer complications. This supports our findings that unilateral laminotomy is a less invasive and more efficient alternative to traditional laminectomy.

In this study, the clinical outcomes for both groups were comparable. Recent data comparing unilateral and bilateral laminotomies indicate that unilateral laminotomy yields slightly superior clinical results in the short term. This discrepancy is likely due to the higher incidence of perioperative complications associated with the technically challenging nature of performing unilateral laminotomy and decompression on both sides. The unilateral approach is performed through a narrow epidural space, and surgeons may face difficulties in adequately decompressing the contralateral side, particularly when undercutting the medial portion of the opposite facet joint. Limited visualization leads to a steep learning curve. However, in this study, both surgeons were highly experienced with each technique, and no technical bias was present. This may explain why perioperative complications were not significantly different between the two groups.

The unilateral approach resulted in less blood loss and a shorter operative time, which correlates with findings in prior reports on unilateral procedures (37, 38). Radiologically, the spinal canal was slightly smaller after unilateral laminotomy compared to the bilateral approach, yet the relief of leg pain was nearly identical between the two groups No patient in the unilateral laminotomy group required further surgery due to incomplete decompression. There were no significant differences in translational motion or disc height between the two approaches.

In our study, ODI scores and VAS for back pain were lower in the unilateral laminotomy group compared to the conventional laminectomy group. This difference was statistically significant (*P* < 0.001), which may be attributed to the reduced damage to paraspinal muscles and facet joints in the unilateral group. The unilateral laminotomy group showed significantly superior results in Back VAS and ODI outcomes, with *P*-values of <0.001 for both measures. This highlights the potential advantages of unilateral laminotomy over conventional laminectomy in terms of pain reduction and functional improvement.

Several minor postoperative complications were observed in the study. Specifically, one patient in the unilateral laminotomy group and one patient in the conventional laminectomy group experienced dural tears, both of which were promptly managed with sutures, and no further complications or morbidities were reported. These minor complications did not have a significant impact on the postoperative pain and functional disability scores, as reflected in the VAS and ODI measures. The absence of major postoperative complications, such as CSF fistula, local/systemic infection, or significant neurological deficits, suggests that both surgical approaches are relatively safe.

While these findings are promising, several limitations should be acknowledged. First, this study was retrospective, and there was no randomization in the group allocation, which could have introduced selection bias. Second, the sample size was relatively small, and the follow-up period was limited to a year. A prospective study with a larger cohort and longer follow-up is necessary to confirm these results. Moreover, the study did not address the long-term effects of both procedures on adjacent segments or the potential for postoperative instability over time. A further limitation of this study is the method of group allocation. Surgical procedures were chosen by two experienced surgeons according to patient preference, rather than randomization, which may have introduced selection bias. Baseline clinical symptoms and radiographic severity of stenosis were comparable between groups. However, psychological factors, not evaluated in this study, could also have influenced postoperative recovery. Future research should include standardized assessments of mental health to improve the reliability of the findings.

In conclusion, both unilateral and conventional laminectomy produce satisfactory clinical outcomes for treating degenerative spondylolisthesis-related spinal stenosis. Unilateral laminotomy, with its reduced blood loss, shorter surgical time, and lower postoperative back pain, is recommended as a less invasive alternative to conventional laminectomy. The radiological data suggest that both procedures provide comparable decompression, making unilateral laminotomy a viable option to minimize the risk of postoperative iatrogenic instability.

## Data Availability

The original contributions presented in the study are included in the article/Supplementary Material, further inquiries can be directed to the corresponding author.
